# Challenges of Improving the Evidence Base in Smaller Surgical Specialties, as Highlighted by a Systematic Review of Gastroschisis Management

**DOI:** 10.1371/journal.pone.0116908

**Published:** 2015-01-26

**Authors:** Benjamin S. R. Allin, Win Hou W. Tse, Sean Marven, Paul R. V. Johnson, Marian Knight

**Affiliations:** 1 National Perinatal Epidemiology Unit, Oxford, United Kingdom; 2 Department of Paediatric Surgery, Oxford Radcliffe University Hospital, Oxford, United Kingdom; 3 Sheffield Children’s Hospital, Sheffield, United Kingdom; The Chinese University of Hong Kong, HONG KONG

## Abstract

**Objective:**

To identify methods of improving the evidence base in smaller surgical specialties, using a systematic review of gastroschisis management as an example.

**Background:**

Operative primary fascial closure (OPFC), and silo placement with staged reduction and delayed closure (SR) are the most commonly used methods of gastroschisis closure. Relative merits of each are unclear.

**Methods:**

A systematic review and meta-analysis was performed comparing outcomes following OPFC and SR in infants with simple gastroschisis. Primary outcomes of interest were mortality, length of hospitalization and time to full enteral feeding.

**Results:**

751 unique articles were identified. Eight met the inclusion criteria. None were randomized controlled trials. 488 infants underwent OPFC and 316 underwent SR. Multiple studies were excluded because they included heterogeneous populations and mixed intervention groups. Length of stay was significantly longer in the SR group (mean difference 8.97 days, 95% CI 2.14–15.80 days), as was number of post-operative days to complete enteral feeding (mean difference 7.19 days, 95%CI 2.01–12.36 days). Mortality was not statistically significantly different, although the odds of death were raised in the SR group (OR 1.96, 95%CI 0.71–5.35).

**Conclusions:**

Despite showing some benefit of OPFC over SR, our results are tempered by the low quality of the available studies, which were small and variably reported. Coordinating research through a National Paediatric Surgical Trials Unit could alleviate many of these problems. A similar national approach could be used in other smaller surgical specialties.

## Introduction

With variation existing around the specifics of management of some of the most common neonatal surgical conditions including oesophageal atresia, gastroschisis and Hirschsprung’s disease, research should be commonplace. Yet despite these fertile conditions for research, for these three conditions, only fourteen prospectively registered clinical trials and two systematic reviews could be identified on a search of the major registries at the time of writing (Table 1). Whilst both the low incidence of many of the studied conditions, and the difficulties in gaining ethical approval are at least in part responsible for the lack of paediatric surgical research, we do not believe that they account for the whole story.

**Table 1:** Number of unique clinical trials/systematic reviews registered for each condition (number with direct surgical relevance).

	**Oesophageal Atresia**	**Gastroschisis**	**Hirschsprung’s Disease**
Clinicaltrails.gov	1 (1)	8 (4)	3 (0)
ISRCTN	0	0	0
International Clinical Trials registry	1 (1)	1 (1)	0
UMIN clinical trials registry	0	0	0
Nederlands Trial Register	0	0	0
Australia New Zealand Clinical trials registry	0	0	0
European Clinical Trials Register	0	0	0
Cochrane database	0	2 (1)	0

Using a systematic review and meta-analysis of gastroschisis as an example, we aimed to highlight some of the key challenges in paediatric surgical research, and suggest ways in which the strength of our clinical evidence base could be improved.

## Background and Aims

Gastroschisis is an increasingly common paediatric surgical condition [1], with an incidence in the United Kingdom of approximately 3.5/10000 births [2]. Multiple surgical repair strategies have been developed, which vary based upon whether general anaesthesia is used, whether the repair is performed as a single stage, and whether closure is operative or non-operative [3–6]. The most commonly employed techniques are operative primary fascial closure (OPFC) and silo placement with staged reduction and delayed closure (SR) [2]. Choice of methodology is usually based upon the operating surgeon’s preference, alongside assessment of viscero-abdominal disproportion and the need or otherwise, to avoid general anaesthesia.

Whilst it is generally accepted that patients with complex gastroschisis (at least one of intestinal perforation, necrosis or atresia that are amenable to immediate operative correction) require an individualized approach to their surgery and generally have a poorer outcome, little consensus has been achieved on the ideal repair strategy for patients with simple gastroschisis (defined as intact continuous bowel that is not compromised or breached at delivery or presentation [7]), and in particular, whether there is a role for routine silo placement with staged reduction and delayed closure.

The aim of this study, therefore, was to perform a systematic review of the literature comparing outcomes of infants with simple gastroschisis following either OPFC or SR. From this study we aimed to identify some of the reasons why research in paediatric surgery is currently largely unable to provide a strong evidence base to inform clinical practice.

## Methods

The review was conducted according to a pre-specified protocol. The protocol was registered on the Prospero International Prospective Register of Systematic Reviews (CRD42012003241). We identified outcome measures associated with complications of gastroschisis repair from previously published literature and expert opinion. Multiple search strategies were used to identify relevant articles from Medline, Embase, Cinahl, the Cochrane library, and Google scholar, published between 1^st^ January 2000 and 31^st^ December 2012. Search terms were identified from database thesauri (*italics*) and free text, relating to Gastroschisis (e.g. *Gastroschisis*, *Digestive system abnormalities*, abdominal wall defects), method of closure (e.g. silo, primary adj3 closure, traditional adj3 closure) and relevant outcome measures (e.g. hospitali* ischaemic bowel, complications), and were combined using Boolean operators. Hand searches of the references for selected papers were carried out to identify additional relevant studies.

All study designs except expert opinion were included, and no restrictions were made on the basis of study language or geographical location. Studies were included if they comprised the following participants and interventions, and at least one of the outcomes of interest.

### Participants

Studies were included if they involved infants born with simple gastroschisis, defined as gastroschisis with an intact continuous bowel that is not compromised or breached at delivery or presentation. Infants with complex gastroschisis, defined as the presence of at least one of intestinal perforation, necrosis, or atresia were excluded from this review, as they only account for approximately 11.5% of infants with gastroschisis, are known to have a worse prognosis than the majority with simple gastroschisis [2], and thus represent a major confounder in any examination of the outcomes of different management techniques.

### Interventions

Correction of gastroschisis can broadly be broken down into two parts; method of visceral reduction, and method of closure. Our intervention of interest was SR, defined as use of a silo (pre-formed, custom or improvised) for staged reduction of the abdominal viscera, followed by any form of defect closure. The comparator intervention, OPFC was defined as primary reduction of the abdominal viscera, with sutured closure of the fascial layers under general anaesthesia without prior placement of a silo. Studies were included if they compared OPFC with SR. Studies including data on only one intervention (i.e. with no comparison group) were specifically excluded.

### Outcome measures

Primary outcome measures:

1. Mortality
2. Length of hospital stay
3. Time to complete enteral feeding/duration of parenteral feeding

Secondary outcome measures:

1. Duration of ventilator use
2. Infective complication
3. Perforation
4. Ischaemic bowel
5. Necrotising Enterocolitis (NEC)
6. Anastomotic stricture
7. Adhesional small bowel obstruction
8. Complications related to stoma
9. Short bowel syndrome
10. Liver disease associated with intestinal failure

### Potential confounding factors

In recognition that other patient factors including prematurity, low birth weight and associated anomalies may also impact on outcome, we sought to describe the distribution of these potential confounders. We did not attempt to control for these amongst the inclusion criteria, as we felt this would be unnecessarily restrictive.

### Study assessment

Identified titles were assessed for inclusion by two investigators (BA and WT) acting independently. Any conflicts were resolved by a third investigator (MK). Data from included articles were extracted independently by the same two investigators (BA and WT), and any differences resolved by discussion. Where required, unpublished data were requested from the authors of each of the included studies to allow for meta-analysis.

### Statistical analysis

Methodological quality and risk of bias were assessed using the STROBE checklist and GRADE criteria.

Data were synthesized using standard methods as described in the Cochrane Reviewers Handbook [8]. The chi-squared test for heterogeneity was used to assess the extent to which the results of the studies were in agreement. Using a conservative cut-off of p<0.01, no statistical heterogeneity was detected and therefore fixed-effect methods were used throughout. Dichotomous outcomes were meta-analysed using Mantel–Haenszel fixed effects methods to produce summary odds ratios with 95% confidence intervals. Continuous variables were meta-analysed using the inverse-variance fixed-effect method to produce weighted mean differences and 95% confidence intervals. All analyses were performed using Review Manager 5.2 (Copenhagen: The Nordic Cochrane Centre, The Cochrane Collaboration, 2012.)

## Results

### Characteristics of included studies

**Figure 1:**
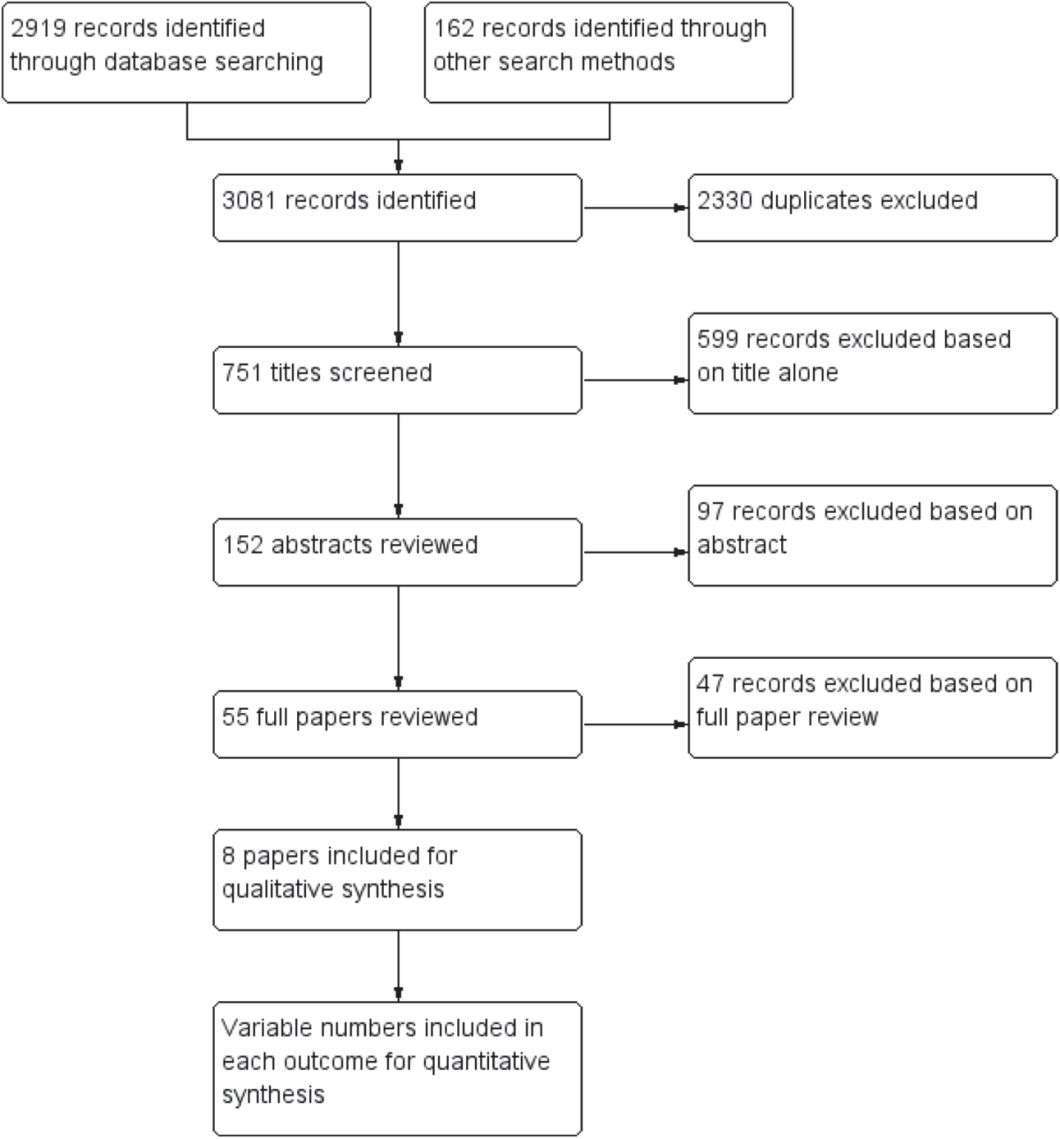
Study flow diagram.

Using the stated search strategy, 3081 papers were identified. After removal of duplicates, 751 unique articles remained. From this, 8 papers were deemed to meet the inclusion criteria, all of which were cohort studies (Fig. 1). No randomised controlled trials were identified that met the inclusion criteria. All papers bar one were published in English language journals. One Spanish paper was translated. Unpublished data were obtained from two authors [2, 9].

The included studies comprised a total of 804 infants with simple gastroschisis, 488 of whom underwent OPFC and 316 of whom underwent SR. The largest study was Owen 2010 [2] with 290 eligible patients. The majority of the remaining studies included fewer than 50 participants (Table 2).

**Table 2:** Summary of Study Characteristics.

**Study**	**Setting and methodology**	n*	Intervention**	**Outcomes**	
				**Primary**	**Secondary**
**Owen 2006 [4]**	Retrospective case-control study of neonates born with gastroschisis and treated at Sheffield Children’s Hospital (England) between 1990 and 2004	48	SR without general anaesthesia, with either sutured or non-sutured closure		Re-operation Post-operative NEC Post-operative infective complications Post-operative stricture formation
**Bonnard 2008 [20]**	Retrospective cohort study of neonates born with gastroschisis and treated at the Hospital for Sick Children in Toronto (Canada) between January 2000 and December 2005.	22	SR with Sutured closure		Post-operative NEC Post-operative small bowel obstruction Post-operative infective complications
**Payne 2009 [9]**	Retrospective cohort study of neonates with gastroschisis treated on the NICU of the Children’s Hospitals and clinics of Minnesota—Minneapolis campus (USA) between 1^st^ January 1990 and 31^st^ December 2007.	128	SR with either sutured or non-sutured closure	Length of Stay	
**Rodriguez 2009 [16]**	Retrospective cohort study of neonates with gastroschisis treated at the Children’s Hospital, National Medical Centre West, Guadalajara City between January 2003 and December 2008	34	SR following failed OPFC. Either sutured or non-sutured closure	Length of stay Post-operative days to complete enteral feeding Mortality	Duration of post-operative ventilation Post-operative infective complications
**Kandasamy 2010 [17]**	Retrospective cohort study of neonates born with gastroschisis in North Queensland (Australia) and treated at the neonatal centre in Townsville between 1988 and 2007.	28	SR when unable to perform OPFC. Either sutured or non-sutured closure	Mortality	Re-operation Post-operative NEC Post-operative infective complications
**Tsai 2010 [19]**	Retrospective cohort study of neonates with gastroschisis treated at Chang Gung Children’s Hospital (Taiwan) between 1996 and 2007	35	SR with either sutured or non-sutured closure	Length of stay Post-operative days to complete enteral feeding	Duration of post-operative ventilation
**Owen 2010^#^ [2]**	Population based cohort study of all infants born with gastroschisis in the United Kingdom and Ireland between October 2006 and March 2008. Patients identified via the BAPS-CASS network.	290	SR with either sutured or non-sutured closure	Length of stay Post-operative days to complete enteral feeding Mortality	Duration of post-operative ventilation Re-operation Post-operative NEC Post-operative infective complications Liver disease associated with intestinal failure
**Bradnock 2011^#^[7]**	One-year follow up data from a population based cohort study of all infants born with gastroschisis in the United Kingdom and Ireland between October 2006 and March 2008. Patients identified via the BAPS-CASS network.	219	SR with either sutured or non-sutured closure	Length of stay	Post-operative small bowel obstruction Post-operative stricture formation Post-operative ischaemic bowel Liver disease associated with intestinal failure Stoma complications

* Number of neonates enrolled in the study who had simple gastroschisis and a comparison of outcomes for SR vs. OPFC

** Study intervention that was used as a comparator to OPFC

^#^ Bradnock 2011 reports one year follow-up data for the patients enrolled in the Owen 2010 study. Both studies are included here as they report different outcomes. However we have only used data from one of the two reports in each outcome analysis.

Although reporting of baseline characteristics was variably done by the included studies, where it was reported, there were no statistically significant differences in birth-weight, gestational age, or associated anomalies between the OPFC and SR groups.

### Characteristics of excluded studies

There were three main types of excluded studies. These were: studies which did not exclude patients with complex gastroschisis, studies which did not directly compare OPFC and SR, and studies which adulterated either the control or intervention group with other closure methods. As a result, we were unable to include several potentially useful studies, including three large retrospective cohort studies based upon data from the Canadian Paediatric Surgical Network (CAPSNet) [10–12] and one randomised controlled trial [6]. See supplementary table for summary of characteristics of excluded studies.

### Key Outcomes

**Figure 2:**
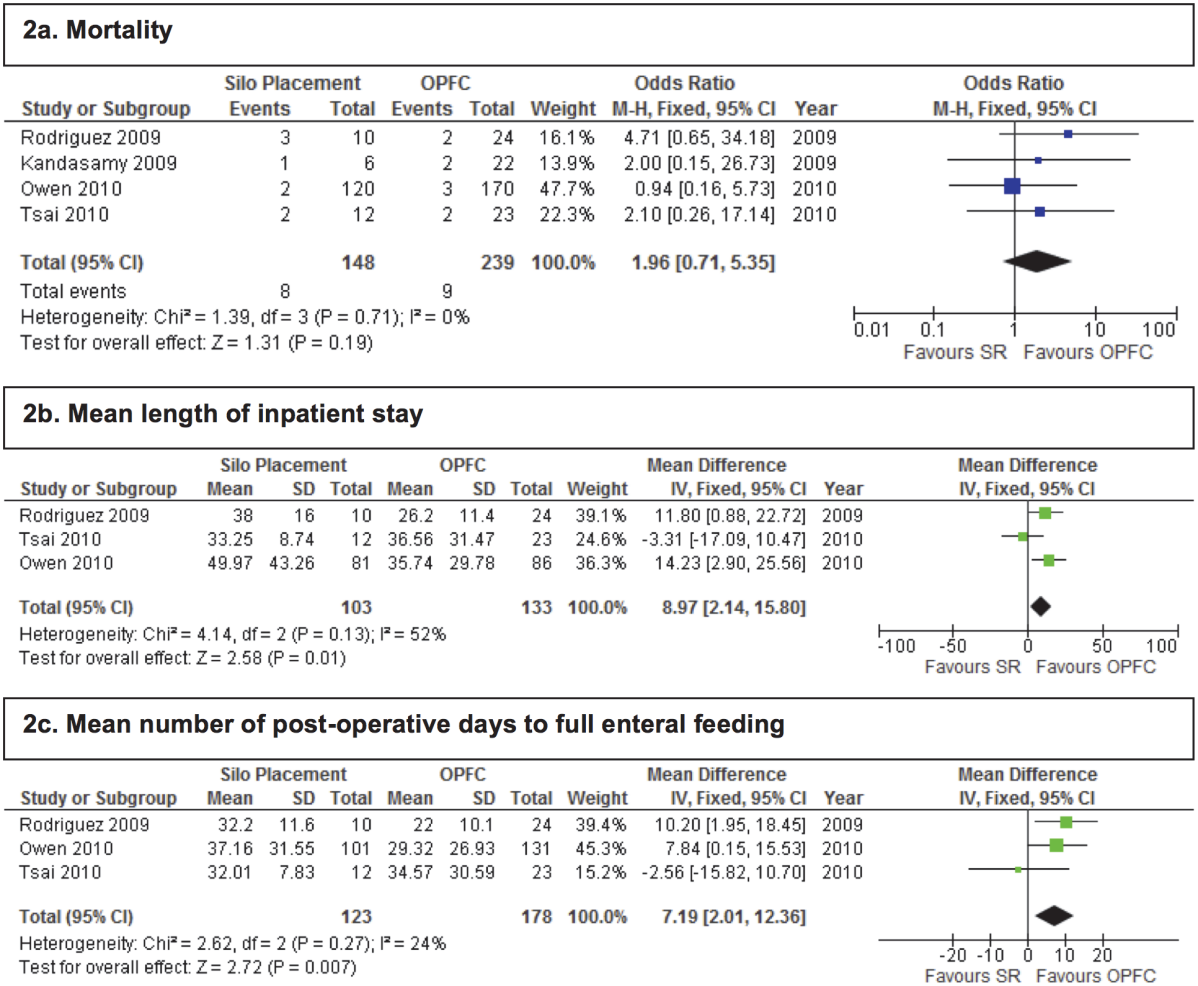
Forest plots showing the effect of SR on primary outcome measures.

**Figure 3:**
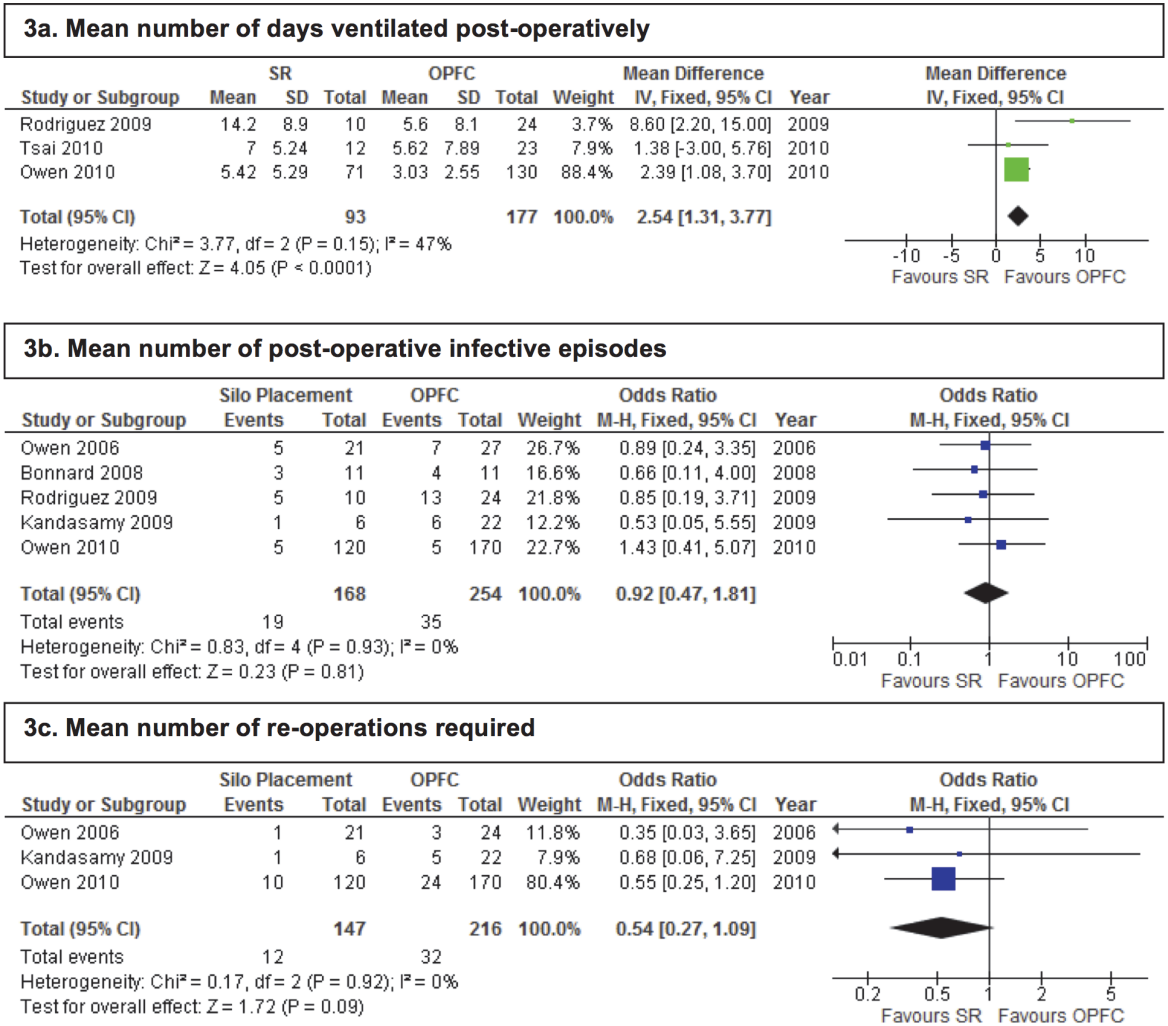
Forest plots showing the effect of SR on key secondary outcome measures.

All three primary outcome measures appeared to favour the OPFC group over the SR group. Both length of stay and number of post-operative days to complete enteral feeding were clinically and statistically significantly longer in the SR group, with a mean difference of 8.97 days, (95% CI 2.14–15.80 days), and a mean difference of 7.19 days (95%CI 2.01–12.36 days) respectively (Fig. 2). Although the odds of death were raised in the SR group (OR 1.96, 95%CI 0.71–5.35), this difference was not statistically significant (Table 3 and Fig. 2). Results for secondary outcome measures are displayed in Table 3 and Fig. 3.

**Table 3:** Summary Outcome Measures for Silo Repair in Neonates with Simple Gastroschisis.

**Outcomes**	**No of Participants (studies)**	**Quality of the evidence (GRADE)**	**Odds ratio (95% CI)**	**Risk/mean difference with SR (95% CI)**
**Mortality**	387 (4 studies)	⊕⊝⊝⊝ **VERY LOW** ^1^, ^2^, due to risk of bias, imprecision	**OR 1.96** (0.71 to 5.35)	**34 more deaths per 1000** (from 11 fewer deaths to 135 more deaths)
**Mean Length of Inpatient Stay**	236 (3 studies)	⊕⊝⊝⊝ **VERY LOW** ^1^ due to risk of bias		The mean length of inpatient stay in the intervention groups was **8.97 days longer** (2.14 to 15.8 days longer)
**Mean Post-Operative Days to Complete Enteral Feeding**	301 (3 studies)	⊕⊝⊝⊝ **VERY LOW** ^1^ due to risk of bias		The mean post-operative days to complete enteral feeding in the intervention groups was **7.19 days longer** (2.01 to 12.36 days longer)
**Post-Operative Infective Complications**	422 (5 studies)	⊕⊝⊝⊝ **VERY LOW** ^1^, ^4^ due to imprecision	**OR 0.92** (0.47 to 1.81)	**10 fewer complications per 1000** (from 68 fewer complications to 87 more complications)
**Mean Duration of Post-operative Ventilation**	270 (3 studies)	⊕⊝⊝⊝ **VERY LOW** ^1^ due to risk of bias		The mean duration of post-operative ventilation in the intervention groups was **2.54 days longer** (1.31 to 3.77 days longer)
**Re-operation**	363 (3 studies)	⊕⊝⊝⊝ **VERY LOW** ^5^ due to imprecision	**OR 0.54** (0.27 to 1.09)	**62 fewer re-operations per 1000** (from 103 fewer re-operations to 11 more re-operations)
**Post-Operative NEC**	388 (4 studies)	⊕⊝⊝⊝ **VERY LOW** ^5^ due to imprecision	**OR 0.8** (0.39 to 1.63)	**18 fewer cases of NEC per 1000** (from 56 fewer cases of NEC to 51 more cases of NEC)
**Post-Operative Small Bowel Obstruction**	241 (2 studies)	⊕⊝⊝⊝ **VERY LOW** ^4^ due to imprecision	**OR 0.8** (0.23 to 2.76)	**9 fewer obstructions per 1000** (from 35 fewer obstructions to 71 more obstructions)
**Post-Operative Stricture Formation**	267 (2 studies)	⊕⊝⊝⊝ **VERY LOW** ^4^ due to imprecision	**OR 3.67** (0.15 to 91.09)	**-**
**Post-Operative Ischaemic Bowel**	219(1 study)	⊕⊝⊝⊝ **VERY LOW** ^4^ due to imprecision	**OR 0.6** (0.05 to 6.74)	**7 fewer cases per 1000** (from 16 fewer cases to 86 more cases)
**Liver Disease Associated With Intestinal Failure**	219 (1 study)	⊕⊝⊝⊝ **VERY LOW** ^4^ due to imprecision	**OR 0.82** (0.31 to 2.16)	**7 fewer cases per 1000** (from 28 fewer cases to 44 more cases)
**Stoma Complications**	99 (1 study)	⊕⊝⊝⊝ **VERY LOW** ^4^ due to imprecision	Not estimable	See comment

* The basis for the **assumed risk** (e.g. the median control group risk across studies) is provided in footnotes. The **corresponding risk** (and its 95% confidence interval) is based on the assumed risk in the comparison group and the **relative effect** of the intervention (and its 95% CI).

**CI:** Confidence interval; **OR:** Odds ratio;

GRADE Working Group grades of evidence **High quality:** Further research is very unlikely to change our confidence in the estimate of effect. **Moderate quality:** Further research is likely to have an important impact on our confidence in the estimate of effect and may change the estimate. **Low quality:** Further research is very likely to have an important impact on our confidence in the estimate of effect and is likely to change the estimate. **Very low quality:** We are very uncertain about the estimate.

^1^ Allocation to SR in Tsai 2010 and Rodriguez 2009 was based upon failure of OPFC, suggesting that an element of selection bias may enter into the analysis.

^2^ Three small studies with lower quality methodology favour OPFC whilst the one large study with more robust methodology favours SR.

^3^ Cumulative sample size is less than the optimal information size (OIS) and the 95% confidence interval for the pooled effect crosses the line of harm, the line of no effect and the line of benefit.

^4^ Cumulative sample size is less than the OIS and the 95% confidence intervals for the pooled data crosses both the line of benefit and line of no effect

## Discussion

Our results would appear to suggest a potential benefit to OPFC over SR. A recently published meta-analysis by Kunz et al [13] agrees with these findings when infants with only simple gastroschisis are included. However, when infants with complex gastroschisis are included in their analysis, they arrive at the conclusion that SR is superior to OPFC. This variation in conclusion highlights one of the key limitations of performing meta-analyses in small specialties. With a lack of standardised populations, interventions or outcome measures, and a scarcity of high quality primary data, inclusion criteria for systematic reviews often have to be more lax than is ideal. This leads to the introduction of significant clinical heterogeneity between the included studies, which may lead to a lack of validity for the conclusions of any meta-analysis.

Although we have attempted to address this potential lack of validity with strict inclusion and exclusion criteria, our conclusion that OPFC is superior to SR must still be tempered by the low quality of the available primary data, and the limitations of our study.

### Study limitations

This study has highlighted three key themes that recur throughout research into paediatric surgery, and which help account for the lack of a robust evidence base for clinical practice. These are; limitations of study type, limitations of methodology, and inconsistency in outcomes and definitions.

As is representative of most research in paediatric surgery [14], our meta-analysis was limited by the fact that the majority of studies available for inclusion were small, retrospective case series or cohort studies. Due to the impact of chance and confounding on the results of these studies, it is impossible to generate robust guidelines based upon them. To produce evidence-based guidelines, the type of study used for assessment of any given intervention should progress as described by the IDEAL recommendations [15] through the use of large cohort studies, to the gold standard randomised controlled trial. Doing so not only increases the weight of the conclusions of the primary research, but also of any meta-analysis including them.

Even with the most robust meta-analysis, it is impossible to remove elements of bias introduced by methodological limitations within the primary research. Often, surgeons who would preferably perform OPFC will instead perform SR if pre-operatively they identify significant viscero-abdominal disproportion. Such surgeon selection means that the SR group may be more likely to contain infants who are sicker or at greater risk of complications. Given that none of the studies included in this review provided a meaningful comparison of the demographics and severity of illness in the OPFC and SR groups, we have been unable to control for any disparity between these groups when drawing our conclusions, introducing a potential bias against SR. Further bias may have been introduced as in many of our included studies, the comparison between OPFC and SR was made when an institution switched from performing OPFC to SR. This may have introduced a degree of bias in favour of OPFC, as institutions had greater experience in this technique than they did for SR, therefore potentially further artificially improving the outcomes in the OPFC group.

Inconsistency in intervention definition between the included studies led to significant clinical heterogeneity within our studied treatment groups, whilst inconsistency in choice of outcome measures and methods of reporting further hampered efforts at meta-analysing the little data that were available. Payne 2009 [9], Rodriguez 2009 [16] and Kandasamy 2009 [17] only performed SR once OPFC had been attempted and failed, whilst Tsai 2010 and Bonnard 2008 included this same group of patients in the OPFC analysis on an intention to treat stratagem. Heterogeneity also existed in the method of skin closure used. Evidence exists to suggest that this as well as the reduction method may affect outcome [18]. Rodriguez 2009, Kandasamy 2010 and Tsai 2010 [16, 17, 19] presented time dependent variables continuously, whilst Bradnock and Owen presented the same variables as categorical data [2, 7]. Outcome measures were also variably reported, with means reported without standard deviations [17], and data presented in such a way as to make it impossible to determine whether means or medians were being reported [20]. Such incomplete data prevents meta-analysis, whilst the clinical heterogeneity reduces the validity of conclusions drawn from any meta-analysis that could be performed.

### The Way Forward

A large majority of the problems described stem from the low case numbers and heterogeneous population encountered by any single institution. We therefore believe that the way to promote evidence-based paediatric surgery is by enhancing collaboration between centres. With only 28 paediatric surgical centres in the United Kingdom and Northern Ireland, achieving co-ordination of research at each of these centres should not be unattainable. A centralised organisation, such as the British Association of Paediatric Surgeons, outside of the influence of any individual surgical centre would provide the ideal starting point for creation of a National Paediatric Surgical Trials Unit. Inviting study proposals and principal investigator applications from each of the contributory units, combined with the necessity for consultants to show contribution to national audit and research in order to attain revalidation could be used as incentives for ensuring the participation of individual centres. With national research co-ordinated through one centre, the possibility of International co-operation is also increased. Developing links with units such as the Canadian Paediatric Surgery Network (CAPSNet)[12] would create the potential for a truly global network of researchers to be developed, with access to a large population base, and the ability to provide truly robust evidence on which to base the clinical practice of paediatric surgery.

A National Paediatric Surgical Trials Unit could act both as a centre to facilitate co-operation between individual surgical centres, and as a research unit in its own right. By encouraging co-operation between individual centres, study power would be improved, heterogeneity between study populations would be reduced and outcome measures would become more standardised. With buy-in and co-operation from each of the individual surgical centres, a National Paediatric Surgical Trials Unit would have the ability to develop an overarching research programme that would allow generation of sufficient quality data to not only evaluate existing practice, but to take new surgical ideas through from initial evaluation to assessment with national randomised controlled trials, and on to large scale review of established practice, as outlined by the IDEAL recommendations [15]. A similar national approach could be used in other smaller surgical specialties.

## Supporting Information

S1 PRISMA Checklist (DOC)
